# Introducing high school students to the Gene Ontology classification system

**DOI:** 10.12688/f1000research.18061.4

**Published:** 2019-08-06

**Authors:** Mehek Dedhia, Kenneth Kohetuk, Wim E. Crusio, Anna Delprato

**Affiliations:** 1 BioScience Project, Wakefield, MA, 01880, USA; 2 Saint Dominic Savio Catholic High School, Austin, TX, 78717, USA; 3 Institut de Neurosciences Cognitives et Intégratives d'Aquitaine (UMR 5287), Pessac, France; 4 University of Bordeaux (UMR 5287), Pessac, France

**Keywords:** gene ontology, high school students, genomics

## Abstract

We present a tutorial that introduces high school students to the Gene Ontology classification system which is widely used in genomics and systems biology studies to characterize large sets of genes based on functional and structural information. This classification system is a valuable and standardized method used to identify genes that act in similar processes and pathways and also provides insight into the overall architecture and distribution of genes and gene families associated with a particular tissue or disease. By means of this tutorial, students learn how the classification system works through analyzing a gene set using DAVID the Database for Annotation, Visualization and Integrated Discovery that incorporates the Gene Ontology system into its suite of analysis tools. This method of analyzing genes is used by our high school student interns to categorize gene expression data related to behavioral neuroscience. Students will get a feel for working with genes and gene sets, acquire vocabulary, obtain an understanding of how a database is structured and gain an awareness of the vast amount of information that is known about genes as well as the online analysis tools to manage this information that is nowadays available. Based on survey responses, students intellectually benefit from learning about the Gene Ontology System and using the DAVID tools, they are better prepared for future database use and they also find it enjoyable.

## Introduction

Genomics is the branch of biology concerned with the study of genes and their functions (see the National Institutes of Health Frequently Asked Questions about Genetic and Genomic Science). Genomics arose from the acceleration of genetic research which was fueled by the development of rapid and affordable DNA sequencing technologies ( Shendure *et al.*, 2017). This opened the door to the sequencing of entire genomes. Presently, the DNA codes for thousands of genomes from diverse species have been sequenced and studied (see the National Center for Biotechnology Information Genome database).

The goals in genomics research are to address all genes and their inter-relationships in order to understand the combined influence on the function of an organism. With this newfound knowledge of the staggering number of genes that make up an organism, the Gene Ontology (GO) classification system was created by the Gene Ontology Consortium to organize genes by their similarities and differences (see Gene Ontology Consortium ‘About’ page). “Ontology” is not a commonly encountered term and there are several definitions that are related to philosophical concepts.

In the context of information science, as described here, “ontology” is concerned with the representation, formal naming and classification system with the purpose of describing the relationship categories and properties of the data. This is similar to Wikipedia which is also based on a controlled vocabulary, categories to group material by like subject matter, and parent-child terms.

The Gene Ontology information is curated, collected, validated, and annotated by the Gene Ontology Consortium in collaboration with their partners which consist of research groups, research communities and other databases (see http://geneontology.org/docs/go-consortium/

This GO classification system provides the scientific community with a structured vocabulary for defining genes ( Ashburner *et al.*, 2000; du Plessis *et al*., 2011; Hastings, 2017; Thomas, 2017). GO terms are commonly used in most, if not all, databases and analysis tools relevant to bioinformatics, systems biology ( Wanjek, 2011), and genomics studies ( du Plessis *et al*., 2011). GO terms are species specific and are updated monthly as biological knowledge is obtained ( Gaudet *et al*., 2017). GO terms describe how a gene functions at the molecular level, its location within the cell, and what biological programs it is involved with. Each GO annotation is associated with an evidence code that is comprised of six categories: experimental evidence, phylogenetic evidence, computational evidence, author statements, curatorial statements, and automatically generated annotations ( http://geneontology.org/docs/guide-go-evidence-codes/)

The importance of the GO term system becomes apparent when analyzing the organization of genomes and coding regions, the distribution of genes involved in specific processes and the conservation of genes across species ( Gaudet *et al*., 2017; https://www.ncbi.nlm.nih.gov/pmc/articles/PMC5821137/). This classification system is also quite powerful when analyzing data from large scale gene expression studies ( du Plessis *et al*., 2011) that consider co-expression data from specific tissues obtained under defined circumstances such as treatment with pharmaceutical agents, or with neurodevelopmental disorders, cancer, or diabetes as examples. GO terms are instrumental for understanding the functions of these genes.

Introducing GO terms and the gene classification system to high school students will bring them up to speed on a commonly used research tool in current genomics methods and expose them to the vast amounts of data that have been derived from genomics and systems biology studies.

In the subsequent sections we show an example of how to extract information about a gene from its associated GO terms and then provide instruction for a practical exercise which will enable students to profile a list of genes using GO terms in the bioinformatics resource DAVID, The Database for Annotation, Visualization and Integrated Discovery. This is a protocol that we teach to our high school student interns when they are evaluating gene expression data for their summer projects ( Crusio *et al*., 2017, see BioScience Project student posters). The student research internship projects are in the context of behavioral neuroscience. Students typically work with gene expression data associated with a specific brain region or brain disorder. As an example for projects related to learning and memory, gene expression data for the hippocampus would be used. Concerning a neurodevelopmental disorder like Schizophrenia, gene expression data for the prefrontal cortex would be considered. There are many online databases that have freely available gene expression data and this could be a way to expand the scope of this tutorial. We use the Allen Brain Atlas for our primary source of gene expression data in the student internship projects.

## Procedure

### Overview of gene function with GO

The overall structure of GO is hierarchical and is based on parent-child terms where the parent term is broader and child term is more specialized.

GO terms group genes according to 3 categories, each of which are considered a distinct ontology: Molecular Function (MF, molecular-level activities performed by gene products), Biological Process (BP, the larger processes, or biological programs accomplished by multiple molecular activities), and Cellular Component (CC, the locations relative to cellular structures in which a gene product performs a function).

**Figure 1.**
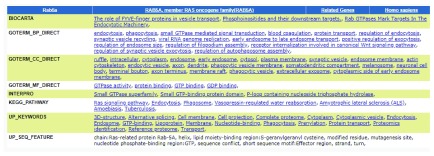
DAVID output for the *RAB5A* gene. Screenshot of the DAVID results for the *RAB5A* gene Top left (blue bar): Gene Symbol identifier. Center (blue bar): full gene name. Labels (left): GO Term BP, GO Term CC, and GO Term MF descriptors. Note that these descriptors are clickable.

As an example, consider the GO term classification for the *RAB5A* gene ( Figure 1). *RAB5A* belongs to a family of genes called RAB GTPases that are key regulators of intracellular membrane trafficking. Rabs are involved in the formation of transport vesicles and their fusion with membranes. They are enzymes and mediate their function by cycling between a GDP bound inactive and a GTP bound active state. Because of their fundamental and ubiquitous role, this family of genes are associated with many biological processes and diseases.

The GO term classification for the *RAB5A* gene gives:

**GOTERM_BP**: endocytosis, phagocytosis, small GTPase mediated signal transduction, blood coagulation, protein transport, regulation of endocytosis, synaptic vesicle recycling, viral RNA genome replication, early endosome to late endosome transport, positive regulation of exocytosis, regulation of endosome size, regulation of filopodium assembly, receptor internalization involved in canonical Wnt signaling pathway, regulation of synaptic vesicle exocytosis, regulation of autophagosome assembly

**GOTERM_CC**: ruffle, intracellular, cytoplasm, endosome, early endosome, cytosol, plasma membrane, synaptic vesicle, endosome membrane, actin cytoskeleton, endocytic vesicle, axon, dendrite, phagocytic vesicle membrane, somatodendritic compartment, melanosome, neuronal cell body, terminal bouton, axon terminus, membrane raft, phagocytic vesicle, extracellular exosome, cytoplasmic side of early endosome membrane.

**GOTERM_MF**: GTPase activity, protein binding, GTP binding, GDP binding

From the *RAB5A* related GO terms, we get the overall impression that this gene encodes an enzyme that is involved in signaling, transport and vesicle dynamics and is associated with cell membranes. How do we arrive at this description?

In this example, the information obtained from the **MF** category is that the protein product of the *RAB5A* gene binds to guanine nucleotides: GTP and GDP (Guanosine tri and di phosphate, respectively) and that it is an enzyme. This is evident by the “GTPase activity” term. Whenever the suffix “ase” is used in the context of a gene or protein, it refers to an enzyme, something that catalyzes a chemical reaction. For the **BP** category, there are several terms associated with intracellular transport, signaling, and endocytosis. Finally, the terms associated with **CC** include endosome and endosome-like organelles (melanosomes, synaptic vesicles, phagocytic vesicles), as well as membrane structures (ruffles, rafts).

### Gene Analysis in DAVID

DAVID is a database with a suite of analysis tools that groups genes based on different criteria related to GO terms. DAVID also links to other databases that contain primary source information like The Gene Ontology as well as complementary information related to pathways and human disease. In this exercise, students will use the sample gene lists (DEMOLIST1 or DEMOLIST2) that are accessible from the DAVID database to see how the Gene Ontology classification partitions a set of genes based on GO Terms. Screenshots and videos are provided for step by step instruction. We also provide a video to instruct students on analyzing a gene list in DAVID obtained from a random gene list generator.

### Protocol

**Figure 2.**
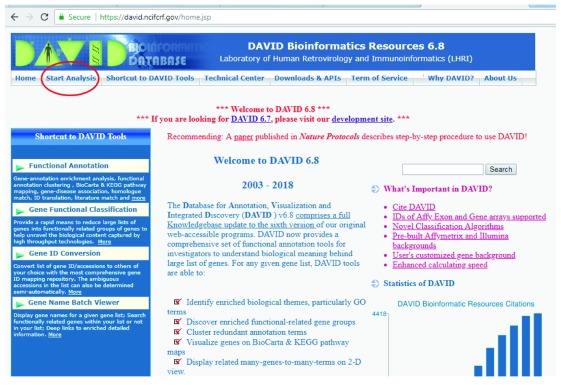
DAVID landing page. Screenshot of the DAVID landing page containing a brief description of the site and links for available tools. The “start analysis” button is circled in red and is located at the top left side of the page. This is the first step in submitting a geneset for analysis with GO Terms in DAVID.

Screenshot 1 ( Figure 2). DAVID landing page. The start analysis link is accessed here and is circled in red in this image. (Video 1, Delprato *et al.*, 2019c)

**Figure 3.**
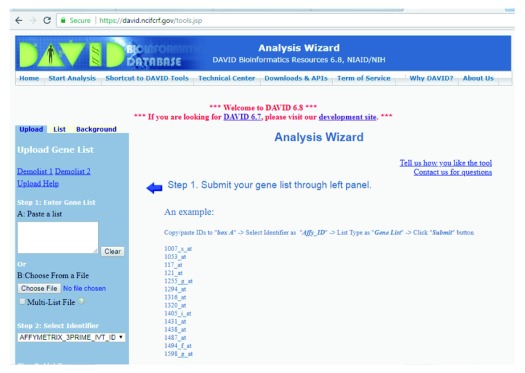
Submitting a geneset. Screenshot of the page where a geneset can be submitted “Step1: Enter Gene List”, there are options to copy and paste a geneset or upload a file. There is also an option to use either of two sample lists provided by the DAVID site. Under the pull down menu, “Step 2 Select Identifier”, there are many types of identification designations for the same gene. For the sample lists, the identifier and species will be recognized automatically. When submitting a gene list from the geneset generator, the identifier is “Official Gene Symbol” which is used in most cases. If the identifier that you choose does not match the identifiers in the submitted gene set, you will receive a message stating this and an option to convert to the correct identifier.

Screenshot 2 ( Figure 3). Submitting a gene list. Select either DEMOLIST 1 or DEMOLIST 2 (left panel). The identifier will come up automatically because this is a demonstration list. If you are submitting your own gene list then, the identifier will have to be specified from the dropdown menu (Video 2, Delprato *et al.*, 2019c). Typically the identifier is the “Official Gene Symbol”. Click “Gene List”, then “Submit List” (Video 1; ( Delprato *et al.*, 2019c)).

**Figure 4.**
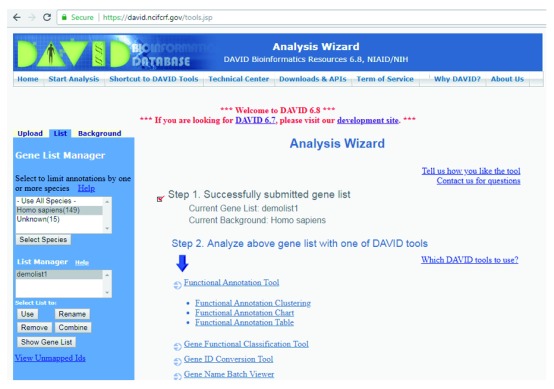
Species selection. Screenshot of a successfully submitted gene set. Here it is necessary to select the species. Again, note that in this instance for the sample genesets provided by DAVID, “Homo sapiens” is already highlighted along with the number of genes that are recognized by the site and for which information is available. Once the species is highlighted, it is necessary to click the “Select Species” button in order to limit the output to just the desired species.

Screenshot 3 ( Figure 4). Species selection. You will see a notice: “Multiple Species, have been Detected”, Highlight “Homo Sapiens” in the window, Select “Homo Sapiens” below the window (Example - DEMOLIST 1: 149 genes, highlighted in grey, left panel). Next, you will see the message “Submission Successful” (Video 1; Delprato *et al.*, 2019c).

**Figure 5.**
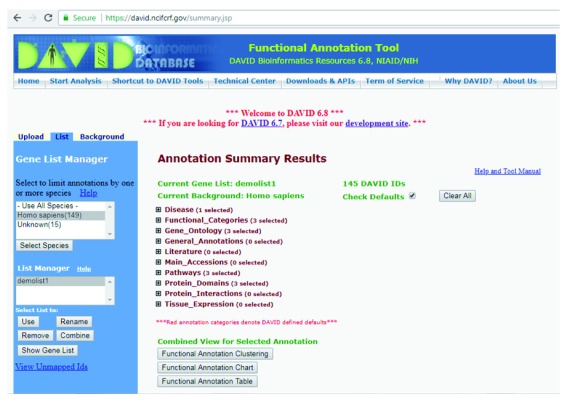
Obtaining the results. Screenshot of the different types of analysis options provided by DAVID for a given geneset. For the purpose of this tutorial, the relevant output is the “Functional Annotation Table” located at the bottom of the page.

Screenshot 4 ( Figure 5). Obtaining the results. Select “Functional Annotation Tool”, beneath the blue arrow. Next, select “Functional Annotation Table”, Bottom of the page (Video 1, Delprato *et al.*, 2019c).

**Figure 6.**
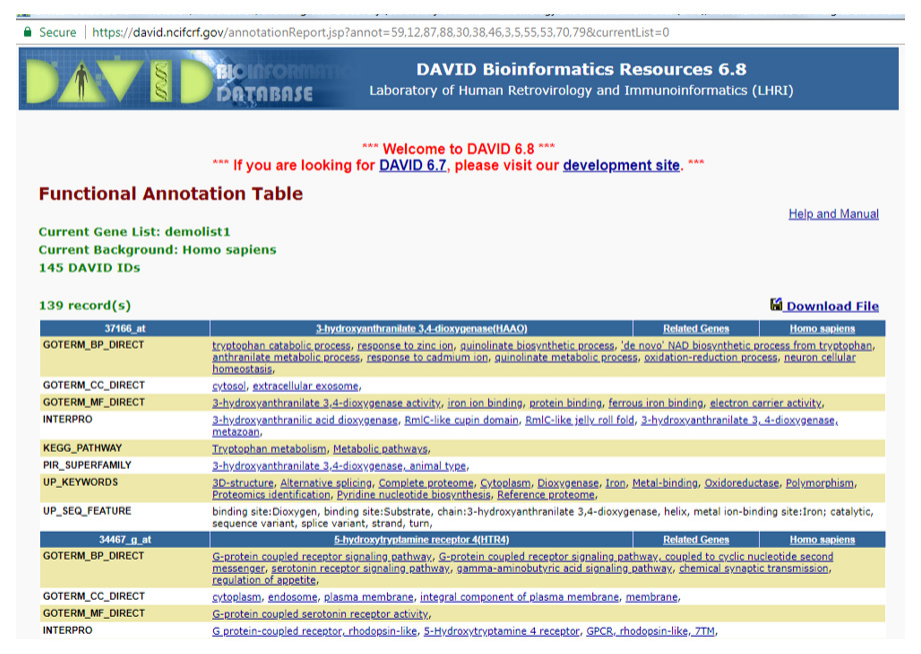
Interpreting the output. Screenshot of the DAVID “Functional Annotation Table” results for a geneset Top left: (blue bar): Gene Symbol identifier. Center (blue bar): full gene name. Labels (left): GO Term BP, GO Term CC, and GO Term MF descriptors. Note that these descriptors are clickable.

Screenshot 5 ( Figure 6). Reading the output. The gene ID and the full gene name are shown in the blue bars above each entry. The GO Term BP (Biological Process), GO Term CC (Cellular Component), and GO Term MF (Molecular Function), terms are clickable descriptors and link to the Gene Ontology website. See above for a complete description of the GO categories (Video 1, Delprato *et al.*, 2019c).

**Figure 7.**
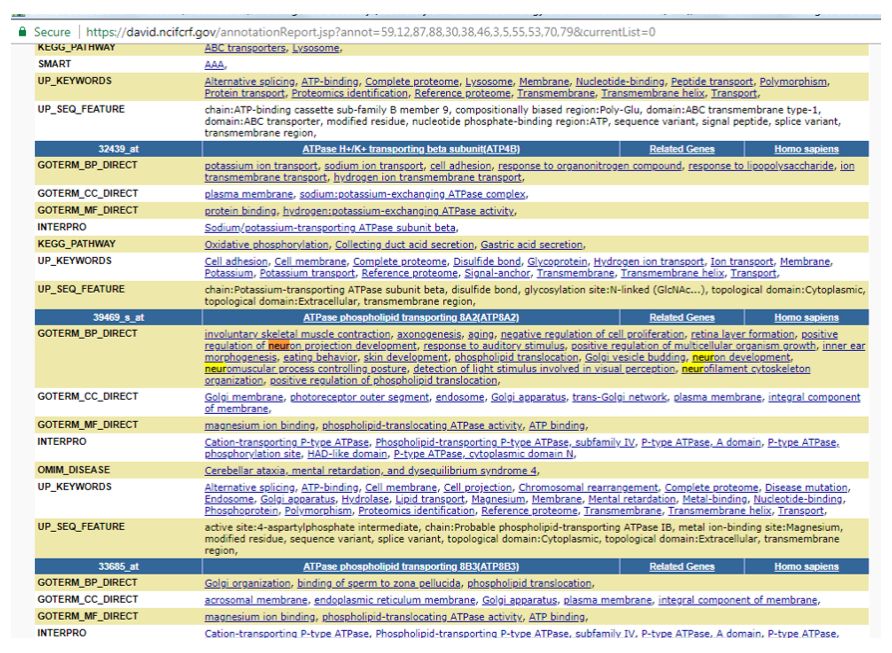
Keyword search. Screenshot of a keyword search of the DAVID output to identify genes that are relevant to a given biological function. In this case the search term is “neur” (highlighted in yellow), to identify genes related to neurological processes. The keyword search is done via the general search function on your computer. For larger gene sets, a computer program may be used.

Screenshot 6 ( Figure 7). Keyword search. When selecting terms for a keyword search, a more complete outcome is achieved if just a few letters are specified. For example, -”neur” will capture terms both starting with neuro and neural (Video 1, Delprato *et al.*, 2019c). DAVID output can be searched for genes related to other process and diseases as well. Have students evaluate the gene list based on their interest. They can identify genes related to a particular process. Students may work individually or in groups.

### Optional exercise

Students may wish to try this with their own gene lists. This online gene list generator will enable students to generate a random list of genes for evaluation. (See also Video 2 for instruction; Delprato *et al.*, 2019c)

### Protocol

Step 1. Specify species: Human is the default

Step 2. Specify list length: 200–500 is a good representative number. Note that DAVID will not evaluate lists with more than 2000 genes. An error message stating this will be received.

Step 3. Select “Generate”

Step 4. Copy the gene list using the “Select All” option and paste the list directly into DAVID for evaluation as described above. Make sure to select “Official Gene Symbol” as the identifier when submitting the gene list.

### Optional exercise 2

**Figure 8.**
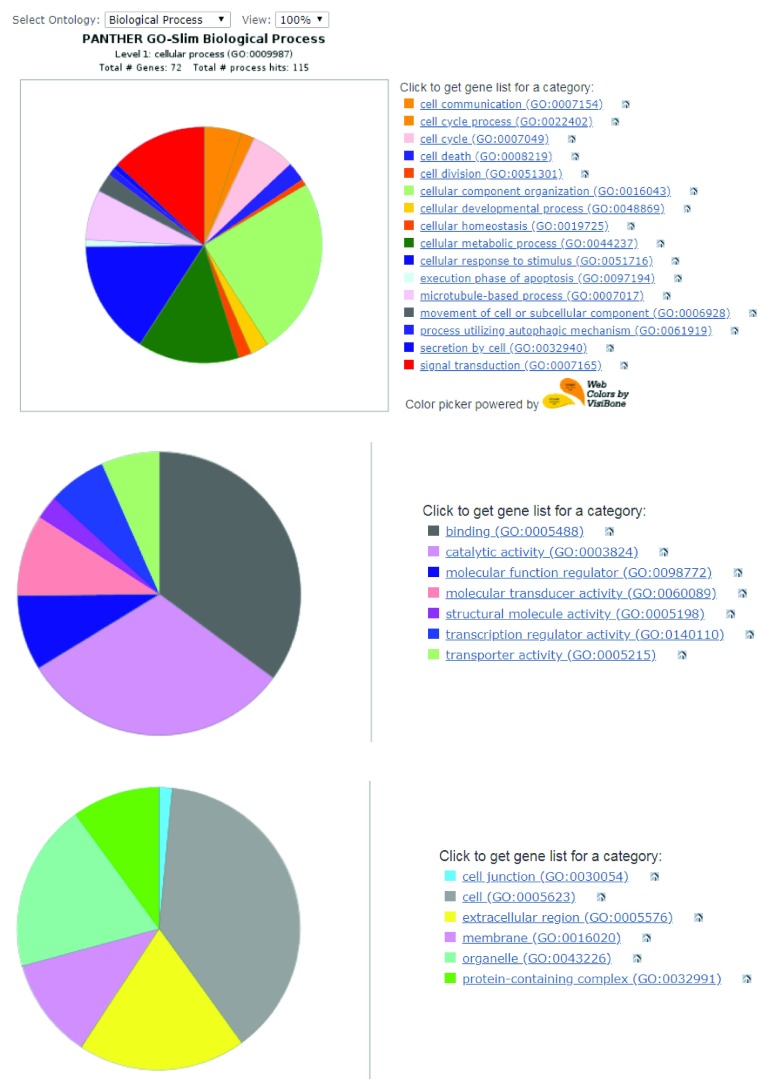
Gene enrichment. Screenshot of a pie chart generated at the Gene Ontology website showing a gene enrichment analysis for a sample gene list along with the associated category legends. The enrichment analysis finds GO terms that are over-represented (or under-represented) for a gene set. In this example, the annotation for Biological Process is shown for 72 genes.

Students may perform a gene enrichment analysis exercise based on GO annotation of their gene list directly at the Gene Ontology site. (Video 3). The results can be viewed as a pie chart which makes for a great visual representation of the GO categories ( Figure 8). Clicking on the individual sections of the pie will result in more inclusive and specific annotation. Clicking on the legends will redirect to a table that contains the gene names associated with a specific GO category (For a more in depth explanation of the results page, see http://geneontology.org/docs/go-enrichment-analysis/).

### Protocol

Step 1. Paste a gene list in the window of the Gene Ontology landing page ( http://geneontology.org/).

Step 2. Specify the ontology (MF, CC, BP) that you would like to analyze from the drop down menu. This can be specified later as well on the results page. (Biological Process is the default).

Step 3. Select species ( *Homo sapiens* is the default).

Step 4. Select “Launch”.

Step 5: Select the clickable number that represents your list of genes relative to the reference list to which is compared.

Step 6. Click on the pie chart above the table and specify the desired ontology from the drop down menu. Pie chart personalization and options for extended information are described in the text above the pie chart. Pie chart and legends may be saved as screenshots.

### Learning assessment

We polled 12 student interns from our summer program for feedback on their experience working with the GO system and DAVID. The survey consists of 5 direct yes or no response questions and two open ended questions. The responses are show in Table 1, Table 2, and Table 3.

**Table 1.** DAVID and GO Student Survey.

Questions	Responses		
	Yes	No	Other
1. Have you ever heard of or did you have any experience working with the DAVID database prior to the summer internship with BioScience Project?	0	12	0
2. Have you ever heard of the Gene Ontology Classification (GO Terms) System prior to the summer internship with BioScience Project?	2	10	0
3. Did you benefit intellectually from working with the DAVID tools and learning about the Gene Ontology System?	12	0	0
4. Did you enjoy working with the DAVID tools and learning about gene analysis with the Gene Ontology System?	12	0	0
5. If applicable, are you better able to navigate other genomic databases as a result of working in DAVID?	11	0	1

**Table 2.** Student Short Answer Responses to question 3a. (Question 3: Did you benefit intellectually from working with the DAVID tools and learning about the Gene Ontology System?) 3a: If yes, please explain how?.

Through this internship using DAVID, I was exposed for the first time to the field of bioinformatics and the gene ontology system. I learned more about the role genes play in neurological disorders in humans. I also got a glimpse of what biomedical researchers and neuroscientists actually do and the type of resources that they work with.
I learned about current methods used by researchers and was able to apply those techniques to my own project. The exposure and experience I gained with bioinformatics gave me a better understanding of gene interactions and how researchers analyze them. The tools I used during the internship furthered my understanding of gene profiling and provided me with insight on the importance of research.
Rather than just learning about genetics as I had in biology classes in the past, I had the opportunity to partake in hands-on learning with the DAVID tools. Through using DAVID and the Gene Ontology system, I gained a broader understanding of the biological functions of genes by seeing and being exposed to such a large variety. Being able to apply what I learned from DAVID and the results I gathered to my own independent project helped to further my understanding of genetics while exposing me to the field of bioinformatics.
I learned different ways genes are classified and analyzed (kegg pathway, etc.). I can use this database for future research projects to get a sense of different pathways these genes are involved in.
I learnt a lot about genomics and how interconnected the different genes in our body are. It also helped me work on my project on Alzheimer’s and the APOE 4 allele.
I was able to learn more about PTSD and identifying candidate genes for PTSD. I performed this project with DAVID and learned a lot about the Gene Ontology System.
The DAVID tools helped me cluster annotation terms based on keywords associated with neurological disorders. Also, the KEGG Pathway map allowed me to visualize genes and how they interact.
This was the first time that I worked with either of these tools and I think I benefited mostly because I got an idea of what working in the field of Biology/Neuroscience would be like.
I was able to learn more about the GO System, different databases such as the Allen Brain Atlas and StringDB, which I used in conjunction with DAVID and learned about how DAVID analyzes the data.
I found that working with the DAVID tools and Gene Ontology System I was exposed to a real world experience in science that gave me a better understanding of where we are in research now, and what is still to be done. The benefits I found in myself were a capital in scientific nomenclature, new skills in analysis of data, and a wholesome exposure into the field of genomics research.
I learned a lot about gene databases and how to look at specific sets of data while ignoring information that may be necessary.
I felt like working with the DAVID tools exposed me to an area of research that I wasn’t previously familiar with in an engaging and fascinating way. Learning how to find specific information about gene interactions/gene functions through an online database is a valuable tool that I believe will benefit me in my future research.

**Table 3.** Student Short Answer Responses to question 4a. (Question 4. Did you enjoy working with the DAVID tools and learning about gene analysis/profiling with the Gene Ontology System?) 4a: If yes, please explain why?.

Working with DAVID and the other databases gave me a chance to learn about the field of bioinformatics and genetics, beyond our school curriculum. It was extremely interesting to learn about the hundreds of genes in the human genome, their various functions both at the molecular and biological level and how they affect the neurological characteristics of human beings. The databases were also very interactive and allowed me to explore the other parts of the database myself.
Although it was initially difficult to navigate the DAVID tools, I found the experience rewarding in the end. The process became easier as I persisted in using the database, and I enjoyed being able to explore the realm of gene profiling.
I enjoyed working with the DAVID tools and learning about gene profiling because from a biological point of view, DAVID is very good at finding relevant information, like other correlates, to the keyword I'm looking for.
I felt like what I was learning about through DAVID and the Gene Ontology system was applicable to my project and I was able to utilize and apply my knowledge of these tools effectively, making me more excited to use it.
I gained insight into gene analysis and gained knowledge that I can use in other situations.
I got practical hands-on experience working with a scientific database which was quite different from the textbook learning taught in schools. I found this quite refreshing and enjoyable.
Although it was pretty confusing for me at the time, I think what made it enjoyable was that I found using these tools interesting especially since this was all new to me! Also it was not too difficult or overwhelming since the provided instructions for the internship walked me through each step. I’m actually now studying Neuroscience at BU and I’m actually hoping to get back to relearning how to use these tools again now that I have a better understanding.
I enjoyed working with the DAVID tools and learning about gene profiling with the GO System because it was very interesting to see how different genes were connected to each other and how far reaching the effects of certain genes are.
Because the breadth and depth of information felt like a million different rabbit holes that I could fall into and learn something new from. However, these tools required navigational help and direction from the supervisor and fellow interns for me to truly reach this point of knowing how to immerse myself in it out of mere curiosity, because of how complex felt at first, and I still have so much more to learn, but overall enjoyed working with these tools once I was comfortable with them.
It was a really interesting and valuable experience to have, and I feel like I learned a lot about how different genes may be connected to each other and what is important to consider and look for in gene profiling.
I thought it was interesting to be able to visualize some of the molecular pathways through the diagrams provided. In completing my research, it was helpful to have all of the biological processes and molecular functions of certain genes all in one place
I really enjoyed working on a topic that was interesting to me. I could learn about PTSD while learning more about biology and gene profiling.

For the direct response questions, 100% of the students had not worked or heard of the DAVID database (Ques1, Table 1.) 83% of the students had not heard of the GO system. Two students had heard of the GO system in an advanced placement biology class but had not explored it further (Ques2, Table1). 100% of the students responded that they benefited intellectually from working with the GO system and DAVID tools (Ques3, Table1) and also that they had enjoyed the experience (Ques 4, Table 1). 92% of the students thought that the experience would better prepare them for future database use. One student indicated that this was not applicable (Quest 5, Table 1).

For the open ended survey questions 3a and 4a ( Table 2 and Table 3), students were asked to explain how they benefited from working with the databases and tools and what about the experience they had enjoyed. A two step process was used to analyze their answers. First, the responses to each question were grouped and an online text analyzer text analyzer was used to assess the words occurring with the highest frequency (Workbooks 1 and 2) ( Delprato *et al*., 2019a; Delprato *et al*., 2019b). Words of 2 or fewer characters were not considered in the analysis. In a subsequent step, a spreadsheet for coding open ended survey questions was used to organize the results from the text analysis (Workbooks 1 and 2).

Response category words chosen represent replies to the question asked. Words containing the same root such as learn, learned, learnt, learning were grouped. The response categories selected for question 3a that may provide insight into why students believe they have benefited intellectually are: expose (5), analyze (4) understand (3), help (3), experience (2), and gained (2) (Workbook 1).

For question 4a, the response categories chosen which may provide insight into why students enjoyed the experience are: interesting (6), gained (2), new (2) and explore (2). Other adjectives used in single responses were: interactive, rewarding, and refreshing (Workbook 2).

### Entry and exit tickets

A basic entry and exit ticket method is suggested to determine what students know about genomics and genes before the lesson as well as what they have learned: main points, questions they may have and what they found most interesting. Sample questions are provided in what follows.

### Entry ticket questions and answers

1. What is a gene?

*A sequence of DNA or RNA which codes for a molecule that has a function.* A gene is the basic physical and functional unit of heredity

2. What is genomics?

*Study of the full set of the genes and DNA in an organism*

3. How many protein coding genes does a human have?

*~20,000*

4. Do humans all have the same genes?

*Yes, but people have different alleles. Alleles are the variation of a gene resulting from mutations. As an example consider eye color. We all have the gene for eye color but some of us have brown, blue or green eyes and there are different shades and hues within those categories.*

5. Do genes work together?
• Yes
6. If yes, provide an example
• Transcription factors
7. Have you ever worked with a biological database?
• Subjective question

### Exit ticket questions

1. What were the main points of the lesson?
2. Do you have any questions?
3. What aspect of this lesson did you find most interesting?
4. Make up a gene and describe it using Gene Ontology classifiers for the 3 categories Molecular Function (MF), Biological Process (BP), and Cellular Component CC)
5. Why do some genes have many classifiers while others do not?
6. For the Biological Process – BP category, what are the classifiers based on, i.e., how are they derived?
7. How do you think you could use this database in a high school research project?

## Conclusions

We describe a procedure for students to become acquainted with the Gene Ontology classification system which is widely used in genomics and systems biology research to characterize gene function. Grouping genes with GO Terms and the DAVID database is based on a protocol that we use with our summer interns to profile gene expression data related to behavioral neuroscience studies ( Crusio *et al*., 2017). Grouping genes in this way identifies genes that function in like processes and also provides information about the overall distribution of a set of genes associated with a particular tissue or process. This tutorial will familiarize early stage students with a biological database and teach them how to mine it and extract useful information from a sample list of genes.

Survey response data from twelve students indicate that they believe they have benefited intellectually from this work and that they enjoy this type of learning experience. Based on coding of the open ended survey responses, the underlying reasons are because they have learned something beneficial and that they find it interesting. The majority of the students (92%) also state that as a result of this experience, they are better prepared for future database use. Entry and exit ticket questions designed to assess student prior and post knowledge as well as stimulate ideas on how these databases and tools may be used in a research project, are included as a formative assessment strategy.

## Data availability

### Underlying data

Figshare: Workbook 1. Coding of student responses to question 3a. https://doi.org/10.6084/m9.figshare.8166611.v1 ( Delprato *et al*., 2019a)

This project contains the following underlying data:

- Coding of student responses to question 3a.xlsx (Coding of responses to open ended student survey question 3a)

Figshare: Workbook 2. Coding of student responses to question 4a. https://doi.org/10.6084/m9.figshare.8166650.v1 ( Delprato *et al*., 2019b)

This project contains the following underlying data:

- Coding of student responses to question 4a.xlsx (Coding of responses to open ended student survey question 4a)

Data are available under the terms of the Creative Commons Attribution 4.0 International license (CC-BY 4.0).

### Extended data

Extended data is available from figshare

Figshare: Extended data 1. Video 1: GeneSetProfiling Analysis Instructional video for using DAVID to obtain Gene Ontology classifiers for a sample geneset which is provided by the DAVID site https://doi.org/10.6084/m9.figshare.8166650.v1 ( Delprato *et al*., 2019c)

Figshare: Extended data 2. Video 2. UploadGeneSet Instructional video for generating a random geneset and submitting this geneset to DAVID for Gene Ontology classification, https://doi.org/10.6084/m9.figshare.7649231.v1 ( Delprato *et al*., 2019d)

Figshare: Extended data 3. Video 3. GeneEnrichment Instructional video for performing a gene enrichment exercise at the Gene Ontology site ( http://geneontology.org/) https://doi.org/10.6084/m9.figshare.9172121 ( Delprato *et al*., 2019e)
