# Supplementary material for: Introducing high school students to the Gene Ontology classification system
Source: F1000Res. 2019 Aug 6;8:241. Originally published 2019 Mar 1. [Version 4] doi: 10.12688/f1000research.18061.4 (PMC6619382; doi:10.12688/f1000research.18061.4)
Supplement: Peer review history

# Peer review: Reviewer response for version 4

**DOI:** 10.5256/f1000research.22130.r52147

**Published:** 2019-08-14

**Authors:** Pascale Gaudet

**Affiliations:** 1 SIB Swiss Institute of Bioinformatics, Geneva, Switzerland

My comments have been addressed.

I confirm that I have read this submission and believe that I have an appropriate level of expertise to confirm that it is of an acceptable scientific standard.

# Peer review: Reviewer response for version 3

**DOI:** 10.5256/f1000research.21308.r50035

**Published:** 2019-07-29

**Authors:** Pascale Gaudet

**Affiliations:** 1 SIB Swiss Institute of Bioinformatics, Geneva, Switzerland

The method presented in this manuscript is of interest to the education community, in that it describes concisely how to use DAVID, a widely-used tool for analysis using GO. However, some points should be addressed to make the method presented here more realistically usable by other schools wishing to introduce this tutorial:

- How are the Gene Ontology, and ontologies in general, introduced to the students? Clearly what is presented in the manuscript is not sufficient. Can the authors provide easily accessible introductory tutorials?
- I am surprised by how much high school students are expected to know about genes and their function, for example when the activity of RAB5A is described. Can the authors provide recommended pre-requisites for this tutorial?
- Likewise, the authors intend to demonstrate how to do a gene profiling, but don't describe what is the purpose of this test.
- Nowhere do authors mention 'annotation' or 'curation'. It is important to explain this concept – how genes come about to be linked to GO terms. 

There are many sentences in the text that are unclear or imprecise:

- The authors state "“Ontology” is not a commonly encountered term and there are several definitions that are related to philosophical concepts.", and then go on to explain only one of these concepts: "In the context of information science, as described here, “ontology” is concerned with the representation, formal naming and classification system with the purpose of describing the relationship categories and properties of the data."
  In this context, I am not clear what the authors mean by a "classification system".
- The importance of the GO term system becomes apparent when analyzing the organization of genomes (...)": As formulated, this information is incorrect.
- Section "Making sense of a gene": that title isn't clear; change title to something along the lines of "Overview of gene function with GO".
- This section doesn't explain how to get this information from the DAVID site. This is essential if this method is meant to be used by other groups.
- Section "Gene profiling in DAVID": No tools on the DAVID website are named "Gene Profiling"; on the DAVID website I get 0 results when I search on "profiling". The authors should either rename the section with the name of the DAVID tool being described, or at the very least explain what they mean by gene profiling.
- The first sentence of that section is "DAVID is primarily a clustering program that groups genes based on different criteria related to GO terms." However, the protocol, demonstrated in Video 1, only shows how to obtain all the functional annotations of all genes from the list inputted, there is no clustering analysis here. The graph that provides clustering is the "Functional Annotation Clustering", but I couldn't get it to work; I get an error message stating that there are too many genes on my list (3000), which by the way was incorrect.
- The authors state that "DAVID links to other databases that contain complementary information like The Gene Ontology". This is incorrect: the Gene Ontology is the source of the data (annotations) presented in DAVID. The Gene Ontology therefore does not contain complementary information; it contains the source data. (Note also that DAVID is much out of date with respect to the GO data; the last update was in 2018, and the previous one in 2009. This should not impact the ability to use this tool for demonstration purposes, but for research project, as those mentioned by the students in their comments, the gene enrichment analysis tool from the GO website should be favored, as its data is updated monthly).
- Screenshot 3 describing Figure 4: The authors state " You will see a notice: “Multiple Species, have been Detected”"; I don't see this text in the page. I do see "Select to limit annotations by one or more species"; maybe this is what the authors are referring to? In that case, for the DEMOLIST sets, there are not multiple species, there is 'human' and 'unknown'. It looks like the 'unknowns' are genes that DAVID cannot map (if you follow the link to Ensembl you will notice it is not there). Students should understand this limitation if they are faced with it: identifiers are unstable, some identifiers may disappear. It's unfortunate that DAVID provides examples containing these unknown identifiers, but if one does the analysis with the 'human list', you are excluding the genes in the 'unknown' list.
- Screenshot 6 describing Figure 7: Keyword search, only demonstrates how to use the built-in functionality of a browser to search text in a web page. While this is good knowledge to have to successfully navigate web-based resources, this is not relevant here.
- There is a mistake in Video 2: When the list is selected from the Random Gene List generator tool, the button 'Copy' should be used, not 'Paste'. In this case, it worked because probably the list was already in the clipboard. Again, this is pretty basic web browsing or even text management knowledge, I don't think it needs to be part of this tutorial.
- There are no answers to Entry ticket questions 5, 6, and 7.

Typos and errors:

- Figure 2 'David' should be DAVID.
- Question 4 Table 1 should be 92% (11/12) and not 96%.
- Lists are inconsistent: sometimes lines end with a period, sometimes not.

I strongly recommend that these points are addressed before the manuscript is indexed.

I confirm that I have read this submission and believe that I have an appropriate level of expertise to confirm that it is of an acceptable scientific standard, however I have significant reservations, as outlined above.

# Response

**Published:** 2019-07-31

**Authors:** Anna Delprato

**Affiliations:** BioScience Project, Wakefield, USA

**Reviewer 4**

 The method presented in this manuscript is of interest to the education community, in that it describes concisely how to use DAVID, a widely-used tool for analysis using GO. However, some points should be addressed to make the method presented here more realistically usable by other schools wishing to introduce this tutorial:

 How are the Gene Ontology, and ontologies in general, introduced to the students? Clearly what is presented in the manuscript is not sufficient. Can the authors provide easily accessible introductory tutorials?

*We use video conferencing, screen sharing, screenshots, and instructional videos to teach our student interns about the Gene Ontology system. The screenshots and videos that we use for teaching are included in this manuscript and the extended data. We would be willing to provide a tutorial geared to instructors via videoconferencing and screen sharing either one on one or in small groups.*

 I am surprised by how much high school students are expected to know about genes and their function, for example when the activity of RAB5A s described. Can the authors provide recommended pre-requisites for this tutorial?

*Students are not expected to have prior knowledge about genes and their function. This is what they learn about in the internship through direct discussion with the instructor and while working on their projects. We do not use pre-requisites for this tutorial.*

 Likewise, the authors intend to demonstrate how to do a gene profiling, but don't describe what is the purpose of this test.

*By gene profiling, we simply mean analysis to understand function. We have changed the word “profiling” to “analyses” for clarification.*

 Nowhere do authors mention 'annotation' or 'curation'. It is important to explain this concept – how genes come about to be linked to GO terms. 

*We agree and as stated above, we have now discussed this point in the introduction.*

 There are many sentences in the text that are unclear or imprecise:

 The authors state "“Ontology” is not a commonly encountered term and there are several definitions that are related to philosophical concepts.", and then go on to explain only one of these concepts: "In the context of information science, as described here, “ontology” is concerned with the representation, formal naming and classification system with the purpose of describing the relationship categories and properties of the data."

 In this context, I am not clear what the authors mean by a "classification system".

*By classification system we mean the organization and retrieval of information. We have attempted to clarify this point by using an analogy with Wikipedia which also uses a structured and controlled vocabulary like GO. We have added this to the introduction.*

 The importance of the GO term system becomes apparent when analyzing the organization of genomes (...)": As formulated, this information is incorrect.

*We apologize, but we fail to see what is incorrect about this phrase*.

 Section "Making sense of a gene": that title isn't clear; change title to something along the lines of "Overview of gene function with GO".

*The section title has been changed.*

 This section doesn't explain how to get this information from the DAVID site. This is essential if this method is meant to be used by other groups. Section "Gene profiling in DAVID": No tools on the DAVID website are named "Gene Profiling"; on the DAVID website I get 0 results when I search on "profiling". The authors should either rename the section with the name of the DAVID tool being described, or at the very least explain what they mean by gene profiling.

*We apologize for the confusion. We have changed the word “profiling” to “analyses” for clarification.*

 The first sentence of that section is "DAVID is primarily a clustering program that groups genes based on different criteria related to GO terms." However, the protocol, demonstrated in Video 1, only shows how to obtain all the functional annotations of all genes from the list inputted, there is no clustering analysis here. The graph that provides clustering is the "Functional Annotation Clustering", but I couldn't get it to work; I get an error message stating that there are too many genes on my list (3000), which by the way was incorrect.

*We apologize for the confusion. We have omitted the word “clustering” from the sentence.*

 The authors state that "DAVID links to other databases that contain complementary information like The Gene Ontology". This is incorrect: the Gene Ontology is the source of the data (annotations) presented in DAVID. The Gene Ontology therefore does not contain complementary information; it contains the source data. 

*We have removed the word “complimentary” from the sentence.*

 (Note also that DAVID is much out of date with with respect to the GO data; the last update was in 2018, and the previous one in 2009. This should not impact the ability to use this tool for demonstration purposes, but for research project, as those mentioned by the students in their comments, the gene enrichment analysis tool from the GO website should be favored, as its data is updated monthly).

*Students do in fact use the Gene Ontology site in addition to DAVID for their research projects. Based on a suggestion made by Reviewer 3, we have now added an optional gene enrichment exercise using the Gene Ontology *tools.

 Screenshot 3 describing Figure 4: The authors state " You will see a notice: “Multiple Species, have been Detected”"; I don't see this text in the page. I do see "Select to limit annotations by one or more species"; maybe this is what the authors are referring to? In that case, for the DEMOLIST sets, there are not multiple species, there is 'human' and 'unknown'. It looks like the 'unknowns' are genes that DAVID cannot map (if you follow the link to Ensembl you will notice it is not there). Students should understand this limitation if they are faced with it: identifiers are unstable, some identifiers may disappear. It's unfortunate that DAVID provides examples containing these unknown identifiers, but if one does the analysis with the 'human list', you are excluding the genes in the 'unknown' list.

*We have attempted to clarify this point in the figure legend.*

 There are no answers to Entry ticket questions 5, 6, and 7.

*We have now provided answers to Entry ticket questions 5 and 6. Question 7, is subjective (Have you ever worked with a biological database?) and we have not included an answer but have indicated that it is a subjective question.*

 Typos and errors:

 Figure 2 'David' should be DAVID.

*Corrected*

 Question 4 Table 1 should be 92% (11/12) and not 96%.

*Corrected*

 Lists are inconsistent: sometimes lines end with a period, sometimes not.

*Corrected*

 ​​​​​​​

# Peer review: Reviewer response for version 3

**DOI:** 10.5256/f1000research.21308.r50598

**Published:** 2019-07-09

**Authors:** Ruth Lovering

**Affiliations:** 1 Functional Gene Annotation, Institute of Cardiovascular Science, University College London, London, UK

This article describes the resources used to teach students about the Gene Ontology and one of the many functional analysis tools that are available. As someone who provides Gene Ontology (GO) annotations and teaches MSc students about bioinformatics resources, I found this article very interesting. I am surprised that high school students were engaged and understood sufficient biology to appreciate the application of GO and DAVID. An aspect not apparently covered by this course is the source of the annotations included in these resources. Biocuration is a poorly appreciated aspect of annotation resources, the majority of scientists have no idea how the data from scientific papers is being summarised and incorporated into annotation files. This is an ongoing process and it would be useful to discuss this as well, for example the similarity of the GO resource with Wikipedia (which also includes GO annotations). Also to point out that the annotation files are not static but updated on a monthly basis. My final question is whether the authors considered using any other tool? DAVID does not regularly update the annotations used in the analysis (last updated in 2016) and it is difficult to see how the same genes are enriched with similar terms. DAVID also has a complicated system of GO FAT etc, which requires the user to understand the ontology structure. But this does help to limit view of the GO terms retrieved. However, the figures only seem to show the list of GO (and other) terms associated with each gene. A figure showing the enriched terms for the full list would be useful.

I confirm that I have read this submission and believe that I have an appropriate level of expertise to confirm that it is of an acceptable scientific standard.

## References

[1] : Improving Interpretation of Cardiac Phenotypes and Enhancing Discovery With Expanded Knowledge in the Gene Ontology. *Circ Genom Precis Med*.11(2) : 10.1161/CIRCGEN.117.001813 e001813 10.1161/CIRCGEN.117.001813 29440116PMC5821137

# Response

**Published:** 2019-07-31

**Authors:** Anna Delprato

**Affiliations:** BioScience Project, Wakefield, USA

**Reviewer 3**

 This article describes the resources used to teach students about the Gene Ontology and one of the many functional analysis tools that are available. As someone who provides Gene Ontology (GO) annotations and teaches MSc students about bioinformatics resources, I found this article very interesting. I am surprised that high school students were engaged and understood sufficient biology to appreciate the application of GO and DAVID.

 An aspect not apparently covered by this course is the source of the annotations included in these resources. Biocuration is a poorly appreciated aspect of annotation resources, the majority of scientists have no idea how the data from scientific papers is being summarised and incorporated into annotation files. This is an ongoing process and it would be useful to discuss this as well, for example the similarity of the GO resource with Wikipedia (which also includes GO annotations). Also to point out that the annotation files are not static but updated on a monthly basis.

 We have now discussed these important points in the introduction of the revised article - version 4.

 My final question is whether the authors considered using any other tool? DAVID does not regularly update the annotations used in the analysis (last updated in 2016) and it is difficult to see how the same genes are enriched with similar terms. DAVID also has a complicated system of GO FAT etc, which requires the user to understand the ontology structure. But this does help to limit view of the GO terms retrieved. However, the figures only seem to show the list of GO (and other) terms associated with each gene. A figure showing the enriched terms for the full list would be useful.

 We use DAVID because the site and its output is well organized and highly informative for students. In addition to the Gene Ontology classification system and its structure as part of the tutorial. In addition to DAVID we use the Gene Ontology site as well.

 We have included an optional gene enrichment exercise along with a step by step video and an additional figure (Figure 8) showing the results of the gene enrichment.

# Peer review: Reviewer response for version 2

**DOI:** 10.5256/f1000research.20696.r47231

**Published:** 2019-05-02

**Authors:** Jennifer A. Ufnar

**Affiliations:** 1 Department of Teaching and Learning, Vanderbilt University, Nashville, TN, USA

The authors have provided some data in a table to show that the students liked the database method and felt that it was helpful in their learning. I feel that the authors need to dig more deeply into the data, though, to explain this to readers. The results and conclusions sections were very short, and did not explain the data in enough detail. As an educator or someone wanting to follow this new method, I would want more data to show that it does work and why/how it is working. Since the results are qualitative, more work with the data is needed to show that the qualitative data actually shows the reader that this method works (i.e. coding of open ended survey responses).

I confirm that I have read this submission and believe that I have an appropriate level of expertise to confirm that it is of an acceptable scientific standard, however I have significant reservations, as outlined above.

# Peer review: Reviewer response for version 2

**DOI:** 10.5256/f1000research.20696.r47230

**Published:** 2019-04-24

**Authors:** William Grisham

**Affiliations:** 1 University of California, Los Angeles (UCLA), Department of Psychology, Los Angeles, CA, USA

All of my concerns have been met, and the article seem fine as it now stands.

I confirm that I have read this submission and believe that I have an appropriate level of expertise to confirm that it is of an acceptable scientific standard.

# Peer review: Reviewer response for version 1

**DOI:** 10.5256/f1000research.19752.r45178

**Published:** 2019-03-26

**Authors:** Jennifer A. Ufnar

**Affiliations:** 1 Department of Teaching and Learning, Vanderbilt University, Nashville, TN, USA

This article presents an interesting way to introduce gene ontologies and related vocabulary to high school students to enhance biological education. While the proposal describes the method in detail, there is no data presented to show that the method actually works. While this is an interesting protocol, it is not a fully fleshed out methods article. The authors will need to present data to show the viability of the method, or resubmit potentially as a protocol. 

 A few more specific comments about the article are listed below:

1. The abstract is worded as more of a proposal rather than a paper. 
2. The protocol seems very much like following a set of directions (not very thought-provoking or inquiry-based), without relating it to the neuroscience. I think the paper would be much stronger if the authors were to relate it back to the behavioral neuroscience projects that are mentioned in the abstract. 

I confirm that I have read this submission and believe that I have an appropriate level of expertise to confirm that it is of an acceptable scientific standard, however I have significant reservations, as outlined above.

# Peer review: Reviewer response for version 1

**DOI:** 10.5256/f1000research.19752.r45177

**Published:** 2019-03-21

**Authors:** William Grisham

**Affiliations:** 1 University of California, Los Angeles (UCLA), Department of Psychology, Los Angeles, CA, USA

This article is aimed at describing an easily searchable database that can be used by students to search by gene ontologies. The point of the article is to provide a guide to use this tool in education at the high school level, but the article could provide a good starting point for college level instructors and even industry trainers. The article describes the underlying question well and chooses a gene, RAB5A, whose function should be readily understood by most biologists.

 The submitted article is timely because the DAVID tool described is not very intuitive. Thus, an easy to follow guide such as the article provides is most welcome. Notably, database tools are becoming more elaborate, and their complexity has created a barrier for effectively use in teaching, particularly at lower levels. The article does a good job of describing this tool, DAVID, in a fashion that will be intelligible to high school and college level students. Lavish illustrations are provided as are links to videos to aid in instruction.

 The use of Entry Ticket and Exit Ticket questions is an excellent pedagogical idea. The questions, however, seem a bit simplistic.

 A bigger problem with the article is that there are no data showing how using the DAVID tool in teaching has produced changes in measurable ways. Do students do better on tests of content knowledge? Are students more sophisticated in their thinking as a function of using the DAVID tool? Are they better able to navigate other genomic tools? Do they simply enjoy the lessons more than with traditional instruction? Many of these questions can be answered with a simple pre- and posttest quiz or even comparing current scores on tests/evaluations to historical scores. This addition would make this article of much more value to educators who could then see if incorporating this tool into their pedagogy is worthwhile.

I confirm that I have read this submission and believe that I have an appropriate level of expertise to confirm that it is of an acceptable scientific standard, however I have significant reservations, as outlined above.
